# Surmounting tumor resistance to metallodrugs by co-loading a metal complex and siRNA in nanoparticles†

**DOI:** 10.1039/d0sc06680j

**Published:** 2021-02-09

**Authors:** Hongzhi Qiao, Lei Zhang, Dong Fang, Zhenzhu Zhu, Weijiang He, Lihong Hu, Liuqing Di, Zijian Guo, Xiaoyong Wang

**Affiliations:** State Key Laboratory of Coordination Chemistry, School of Chemistry and Chemical Engineering, Nanjing University Nanjing 210023 China qiaohz@njucm.edu.cn; Jiangsu Key Laboratory for Functional Substance of Chinese Medicine, Jiangsu Engineering Research Center for Efficient Delivery System of TCM, School of Pharmacy, Nanjing University of Chinese Medicine Nanjing 210023 China; State Key Laboratory of Pharmaceutical Biotechnology, School of Life Sciences, Nanjing University Nanjing 210023 China boxwxy@nju.edu.cn

## Abstract

Copper complexes are promising anticancer agents widely studied to overcome tumor resistance to metal-based anticancer drugs. Nevertheless, copper complexes *per se* encounter drug resistance from time to time. Adenosine-5′-triphosphate (ATP)-responsive nanoparticles containing a copper complex CTND and B-cell lymphoma 2 (Bcl-2) small interfering RNA (siRNA) were constructed to cope with the resistance of cancer cells to the complex. CTND and siRNA can be released from the nanoparticles in cancer cells upon reacting with intracellular ATP. The resistance of B16F10 melanoma cells to CTND was terminated by silencing the cellular Bcl-2 gene *via* RNA interference, and the therapeutic efficacy was significantly enhanced. The nanoparticles triggered a cellular autophagy that amplified the apoptotic signals, thus revealing a novel mechanism for antagonizing the resistance of copper complexes. In view of the extensive association of Bcl-2 protein with cancer resistance to chemotherapeutics, this strategy may be universally applicable for overcoming the ubiquitous drug resistance to metallodrugs.

## Abstract 2

Bcl-2-related tumor resistance to anticancer drugs can be overcome by silencing the cellular Bcl-2 gene *via* RNA interference. The realization of the goal is exemplified by delivering Bcl-2 siRNA and a tumor-resistant Cu complex to cancer cells with an ATP-responsive nanocarrier.

## Graphical abstract

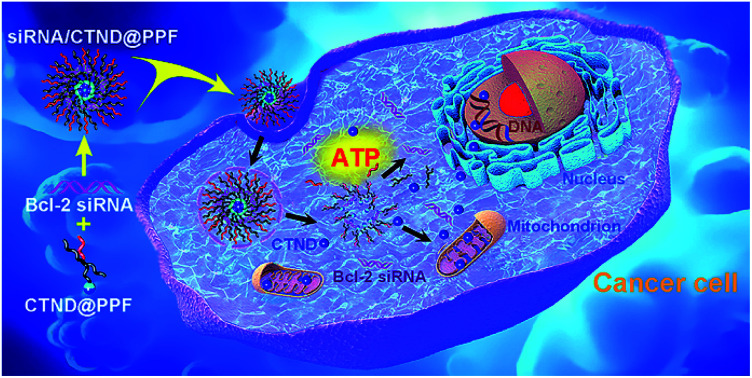

## Introduction

**Fig. 1:**
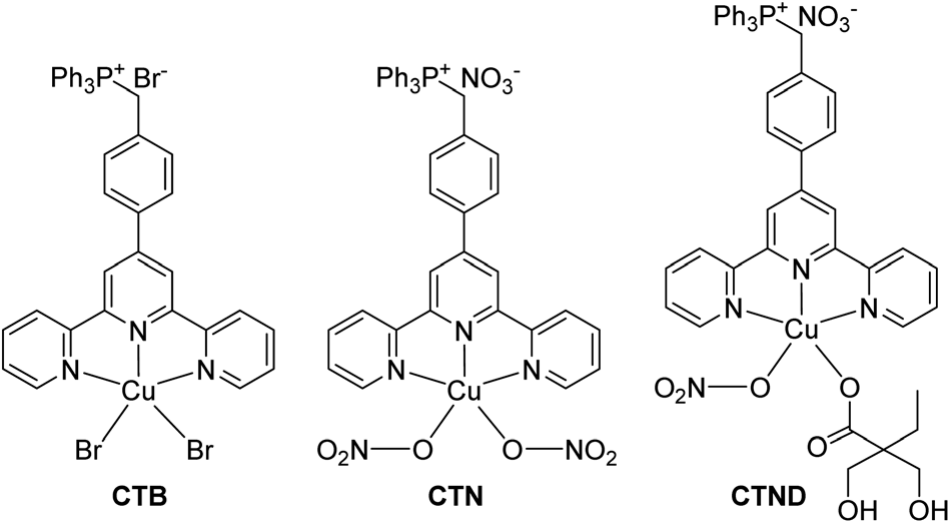
Chemical structures of CTB, CTN and CTND.

Metal-based anticancer drugs represented by cisplatin are the main chemotherapeutics; however, the clinical use of these drugs is largely limited by dose-limiting side effects and drug resistance.^1^ Particularly, drug resistance is a common challenge that metallodrugs have to face. Although the mechanism behind drug resistance is not fully understood, accumulating evidence suggests that it is related to the defective apoptotic signaling,^2^ which is a hallmark of most tumors and contributes to the acquired resistance. Copper complexes are potential anticancer drugs with relatively low systemic toxicity; more importantly, they are expected to overcome the drug resistance encountered by platinum-based drugs *via* a different mechanism of action.^3^ Previously we reported a mitochondrion-targeted copper complex CTB (Fig. 1), and subsequently studied its antitumor activity and mechanism of action.^4^ CTB is more cytotoxic than cisplatin against various tumor cells and especially the cisplatin-resistant ones. Inspired by the encouraging results, we synthesized an analogous copper complex CTN (Fig. 1), which bears a close resemblance to CTB except that two Br^−^ ligands were replaced by two NO_3_^−^ ligands. Unfortunately, CTN encountered drug resistance this time in B16F10 melanoma cells (Fig. S1A†), with its inhibition activity being significantly lower than that of CTB. Fortunately, we found out the reason behind the resistance. In the CTN-treated B16F10 melanoma cells, the B-cell lymphoma 2 (Bcl-2) pro-survival protein was overexpressed, and the Bax proapoptotic protein was down-regulated (Fig. S1B†). It is known that intrinsic apoptosis is regulated by the Bcl-2 protein family which includes pro-survival and proapoptotic subgroups.^5^ Bcl-2 pro-survival protein has been implicated in tumor survival and drug resistance, and its overexpression is positively correlated with resistance of many malignancies, including melanoma.^6^ These findings suggest that Bcl-2 pro-survival protein is involved in the resistance of B16F10 to CTN.

Specific small interfering RNA (siRNA) can target and silence nearly any gene of interest, including genes encoding proteins involved in the acquisition of multidrug resistance.^7^ We have verified this by adding Bcl-2 siRNA to the CTN-treated B16F10 cells, which markedly enhanced the antiproliferative activity of CTN (Fig. S1C†). However, naked siRNA cannot readily cross the biomembranes and is susceptible to degradation by endogenous enzymes.^8^ Therefore, great effort has been made to develop smart and safe vehicles to facilitate the co-delivery of siRNA and chemotherapeutics into cells,^9^ which would colocalize them in tumor regions with the same pharmacokinetic profiles to modulate the drug resistance. In fact, co-administration of siRNA and chemotherapeutics has been expected to achieve optimal therapeutic outcomes through targeting different pathways in tumorigenesis.^10^

Herein CTND (Fig. 1) derived from CTN was used as a tumor-insensitive model copper complex to test the applicability of the siRNA-strategy in overcoming tumor resistance. CTND was co-loaded with Bcl-2 siRNA in tumor-targeted nanoparticles (NPs) to achieve a synergistic effect. A variety of experiments proved that this method is an effective approachh to conquer the Bcl-2-regulated drug resistance to metallodrugs.

## Results and discussion

### Design and preparation

**Scheme 1:**
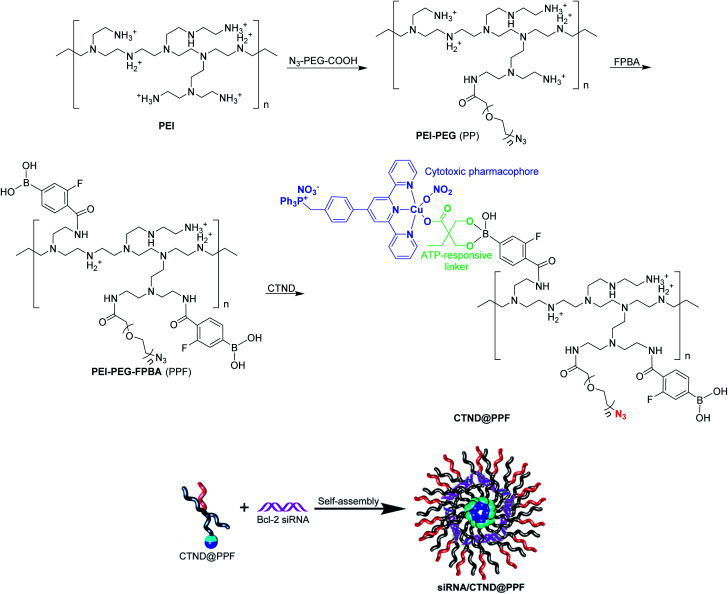
Preparation of siRNA/CTND@PPF nanoparticles.

In order to load CTN more effectively and release it more readily in cancer cells, it was modified with dimethylolbutyric acid to obtain CTND, which could easily react with 3-fluoro-4-carboxyphenylboronic acid (FPBA). CND, an analog of CTND without the targeting group triphenylphosphine (TPP), was also synthesized as a reference compound (Scheme S1†). We intend to use cationic polyethyleneimine (PEI) to shield the Bcl-2 siRNA, because PEI could condense siRNA through positive charges and protect it from nuclease degradation. To form more stable and biocompatible NPs, PEI was modified with azide(polyethylene glycol)carboxylic acid (N_3_–PEG–COOH) to obtain PEI–PEG (PP), which was further modified with FPBA to obtain PEI–PEG–FPBA (PPF). The FPBA residue in PPF could efficiently combine CTND through forming a boronate ester coordination bond to obtain CTND@PPF. The co-loading of CTND and Bcl-2 siRNA in the NPs occurred through coordination-driven assembly and electrostatic condensation, respectively (Scheme 1).

The existance of an azide group (N_3_^−^) on the surface of NPs could facilitate possible further modification by tumor-targeting groups such as iRGD-alkyne peptide *via* click chemistry;^11^ and the boronate ester bond would help ATP release CTND and siRNA (*vide infra*). Scrambled siRNA (siRNASCR, negative control) was subsequently assembled with PPF, CTND@PPF and CND@PPF at different molar ratios of primary amino groups in PEI moiety to phosphate groups in siRNA (N/P ratios) under neutral conditions to obtain siRNASCR@PPF, siRNASCR/CTND@PPF, and siRNASCR/CND@PPF NPs, respectively.

### Characterization

CTND, CND and the corresponding ligands were characterized by electrospray ionization mass spectrometry (ESI-MS) and ^1^H NMR spectroscopy (Fig. S2–S4†). The measured molecular weights well matched with the calculated values, and the representative peaks were identified. The competitive binding of CTND or CND to FPBA in the presence of Alizarin Red S (ARS) showed that the emission fluorescence at 571 nm (an indicator of ARS binding to FPBA) dramatically decreased with the addition of the copper complex (Fig. S5†), manifesting that CTND or CND was connected to the FPBA moieties through coordination bonds.^12^

Intermediate PEG–PEI (PP) and the final product PPF were characterized by ^1^H- and F^19^ NMR, as well as gel permeation chromatography (GPC) analyses (Fig. S6–S8†). ^1^H NMR results indicate that each PEI bound with 15 PEG chains and 10 FPBA molecules. At this substitution degree, PEG could effectively shield the positive charges of PEI and prevent the NPs from being engulfed by the reticuloendothelial system (RES).^13^

**Fig. 2:**
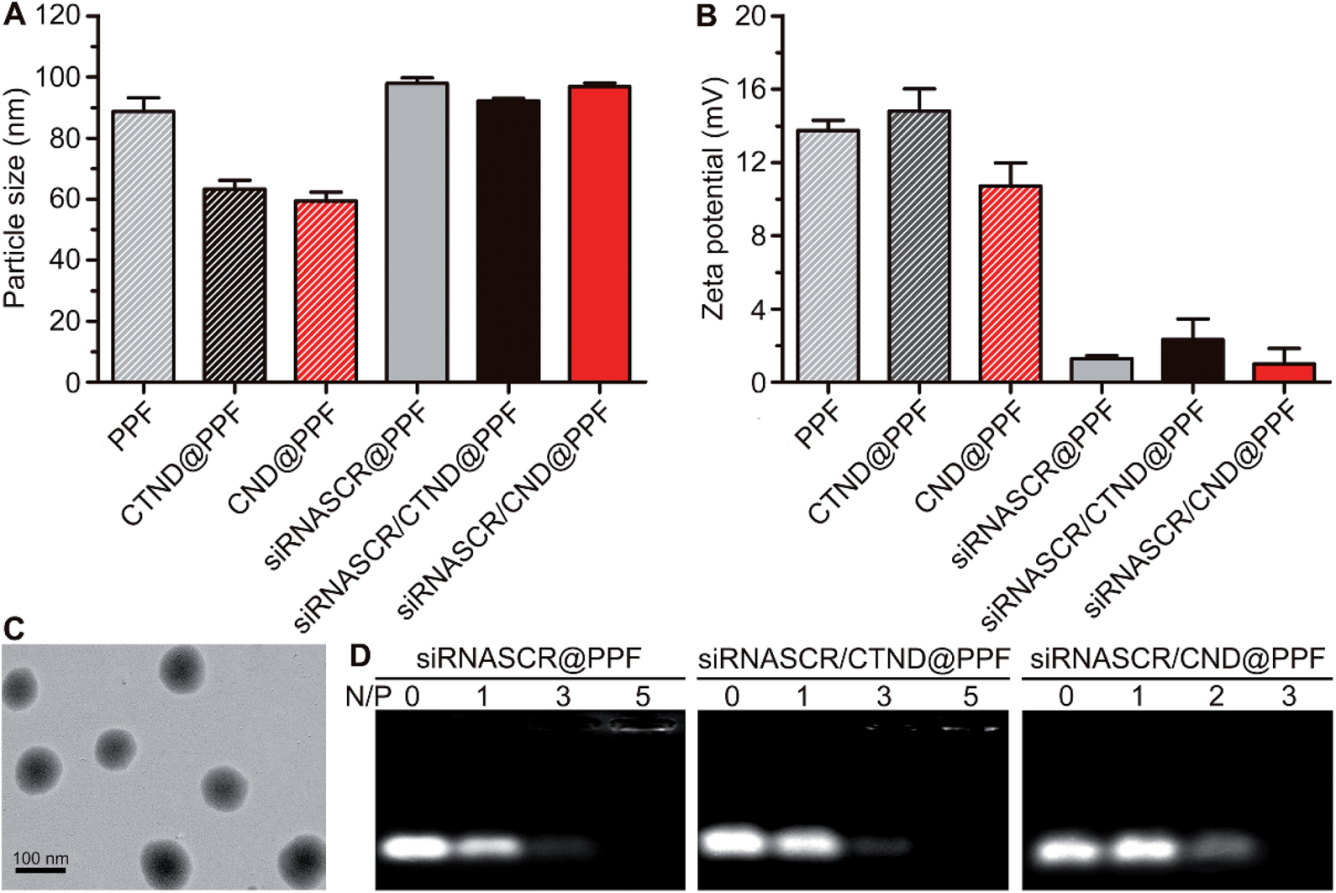
Characterization of different composites. (A) Particle sizes determined by DLS; (B) *ζ* potentials; (C) TEM image of siRNASCR/CTND@PPF stained by 1% phosphotungstic acid; (D) electrophoretic patterns at different N/P ratios.

CTND@PPF and CND@PPF NPs were characterized by GPC analysis. The NPs displayed a unimodal molecular weight distribution and narrow polydispersity index (Fig. S8†), and were stable in HEPES buffer (HB, 10 mM, pH 7.4) or culture medium (Fig. S9†). The drug-loading capacity of CTND@PPF and CND@PPF measured by HPLC was 6.91% and 6.27%, respectively, in terms of CTN. The particle size of all the formulations measured by dynamic light scattering (DLS) was smaller than 100 nm, with a narrow size distribution (Fig. 2A), which would be more conducive to the accumulation of NPs in tumor cells than the larger size.^14^ The *ζ* potential of the NPs decreased significantly after inclusion of siRNASCR (Fig. 2B), which was attributed to the strong negative charges of siRNASCR. Transmission electron microscopy (TEM) images showed that siRNASCR/CTND@PPF was uniform and spherical in shape (Fig. 2C). The optimal N/P ratio was screened using a 1% agarose gel electrophoresis assay. For siRNASCR@PPF and siRNASCR/CTND@PPF, free siRNASCR bands were not detected above the N/P ratio of 5 (Fig. 2D), which appeared to be the threshold for PPF to fully neutralize the negatively charged siRNASCR. The optimal N/P ratio for siRNASCR/CND@PPF was 3. The protection of siRNA by the NP from endonucleases was assessed in fetal bovine serum (FBS). Naked siRNASCR was almost completely degraded after incubation for 4 h, while siRNASCR under the protection of PPF remained intact for 24 h at 37 °C (Fig. S9†). The MTT assay showed that siRNASCR@PPF NPs are almost not cytotoxic to B16F10 cells even siRNASCR at a dose up to 800 nM, confirming that PPF is a biocompatible vector (Fig. S10†).

### ATP-responsive drug release

**Fig. 3:**
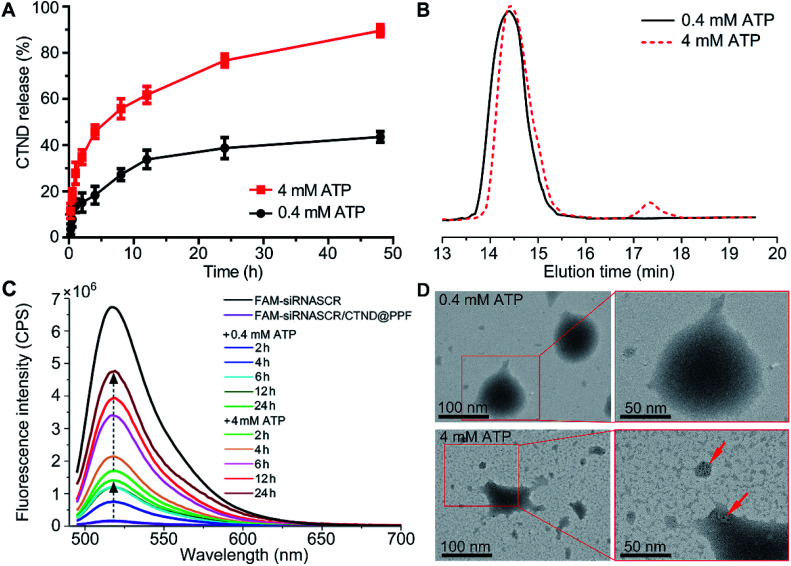
(A) ATP- and time-dependent release of CTND from siRNASCR/CTND@PPF; (B) GPC curves of CTND@PPF at different ATP concentrations after 4 h incubation; (C) fluorescence spectra of FAM-siRNASCR released from FAM-siRNASCR/CTND@PPF; (D) TEM images of siRNASCR/CTND@PPF after treatment at 0.4 and 4 mM ATP (red arrows indicate the aggregated CTND).

ATP is ubiquitous in live cells and extracellular environments, and a great concentration gap exists between an extra- (<0.4 mM) and intracellular (1–10 mM) environment,^15^ which could be used to stimulate the release of the copper complex and siRNA enclosed in the NPs. The release of CTND was tested at 0.4 and 4 mM ATP, which are typical extra- and intracellular ATP concentrations, respectively, to examine the ATP-responsive characteristic of siRNASCR/CTND@PPF NPs. As shown in Fig. 3A, in the 0.4 mM ATP medium, *ca.* 10% of CTND was released in the first 4 h, and the release increased to *ca.* 30% in 24 h. In the 4 mM ATP medium, the release of CTND dramatically accelerated, reaching *ca.* 40% in the first 4 h and more than 70% in 24 h. Moreover, GPC profiles showed that the CTND peak appeared at 17.5 min at 4 mM of ATP, whereas no obvious CTND peak was detected at 0.4 mM of ATP (Fig. 3B). The release of CND from siRNASCR/CND@PPF NPs also showed similar characteristics (Fig. S11†). The release characteristics of siRNASCR were further estimated by comparing the fluorescence intensity of fluorescein amidite (FAM)-labeled siRNASCR released from the FAM-siRNASCR/CTND@PPF NPs. As shown in Fig. 3C and S11,† the fluorescence intensity at 4 mM ATP is much greater than that at 0.4 mM (<15% of FAM-siRNASCR fluorescence at 24 h). The morphological changes of the siRNASCR/CTND@PPF NPs were also observed by TEM. As shown in Fig. 3D, the NPs remained spherical with an overall homogeneous morphology at 0.4 mM of ATP, indicating their enough stability at a low ATP concentration; at 4 mM ATP, the structure of the NPs completely collapsed and some dark spots appeared, presumably resulting from the aggregation of CTND. The results suggest that increased concentrations of ATP could promote the release of CTND and siRNASCR. Several reasons may account for the ATP-dependent release. First, the vicinal diol in ATP could compete with the FPBA-bound CTND, leading to the detachment of CTND; second, the negatively charged ATP could neutralize the positive charges on PEI, reducing the electrostatic attraction to siRNASCR; third, ATP is a biological hydrotrope, which may prevent or dissolve the formation of macromolecular aggregates at millimolar concentrations, causing structural rearrangement and drug leakage.^16^ Thus, by making use of the concentration gradient of ATP between extra- and intracellular environments, siRNASCR/CTND@PPF NPs could safely deliver CTND and siRNA into cells. After further modification with tumor-targeting groups as we mentioned above, such NPs could not only specifically transport the metal complex and siRNA to tumor cells, but could also guarantee their release in an intracellular environment.

The intracellular ATP-dependent release of CTND was estimated by using inductively coupled plasma mass spectrometry (ICP-MS). The endocytosed siRNASCR/CTND@PPF exhibited a sustained release of CTND and lasted for over 6 h (Table S1†). However, the release was significantly inhibited by the addition of iodoacetic acid (IAA) or the cryogenic temperature (4 °C) because of the suppression on the production of ATP (Fig. S12†).^15^ The results indicate that the release of CTND from siRNASCR/CTND@PPF is accelerated by ATP in tumor cells.

### Cellular uptake and distribution

**Fig. 4:**
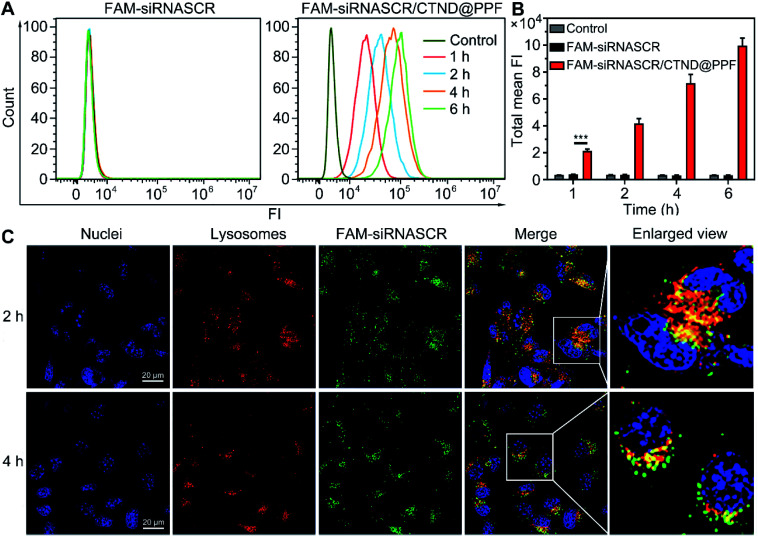
Cellular uptake and distribution of FAM-siRNASCR and FAM-siRNASCR/CTND@PPF in B16F10 cells. (A) Flow cytometry analysis of the cells after incubation for different time periods; (B) quantification of cellular internalization in terms of total mean fluorescence intensity (FI), ***p < 0.001; (C) CLSM images of the endosomal escape of siRNASCR/CTND@PPF. Cell nuclei were stained with DAPI, and the late endosomes and lysosomes were stained with LysoTracker™ red.

The cellular uptake and distribution of siRNASCR/CTND@PPF were evaluated by flow cytometry and confocal laser scanning microscopy (CLSM). In comparison with the naked FAM-siRNASCR, the time-dependent increase of fluorescence intensity indicate that FAM-siRNASCR/CTND@PPF enhanced the uptake of siRNASCR in B16F10 cells (Fig. 4A and B). The distribution of siRNASCR in B16F10 cells at different incubation intervals showed that FAM-siRNASCR (green) was localized within LysoTracker™ red-stained organelles at 2 h, implying that siRNASCR was localized in endolysosomes. After incubation for 4 h, the red and green fluorescence dissociated, suggesting the endolysosomal escape of siRNASCR (Fig. 4C), which was attributed to the “proton sponge effect” of PEI.^17^ The cellular uptake of CTND in B16F10 cells was quantified by ICP-MS. As shown in Table 1, the Cu content in the cells treated with CTND@PPF or siRNASCR/CTND@PPF for 4 h was much higher than that in the cells treated with CTND. Moreover, the ratio of mitochondrial Cu to that of cellular Cu was higher for CTND than for CND due to the mitochondrion-targeting group of TPP. The difference was further enlarged when the complexes were incorporated into the NPs; for example, the value for siRNASCR/CTND@PPF was *ca.* 2.4 times higher than that for siRNASCR/CND@PPF. These results indicate that the mitochondrion-targeting ability of CTND was retained and even improved after inclusion in the NPs. The Cu content directly reflected the drug delivery efficiency and pathway of the NPs.

**Table 1:** Cu contents in B16F10 cells and the mitochondria (Mito) determined by ICP-MS analysis (mean ± SD, *n* = 3)

Complex	Cell (ng mg^−1^)	Mito (ng mg^−1^)	Mito/cell (%)
CTND	84.0 ± 1.7	8.3 ± 2.5	9.9
CND	62.4 ± 1.4	3.1 ± 1.3	5.0
CTND@PPF	145.6 ± 2.8	42.5 ± 1.5	29.2
CND@PPF	101.2 ± 1.1	12.3 ± 1.0	12.2
siRNASCR/CTND@PPF	177.0 ± 0.8	56.6 ± 2.1	32.0
siRNASCR/CND@PPF	140.0 ± 1.2	18.56 ± 1.4	13.3

### Silencing of the Bcl-2 gene and apoptosis

**Fig. 5:**
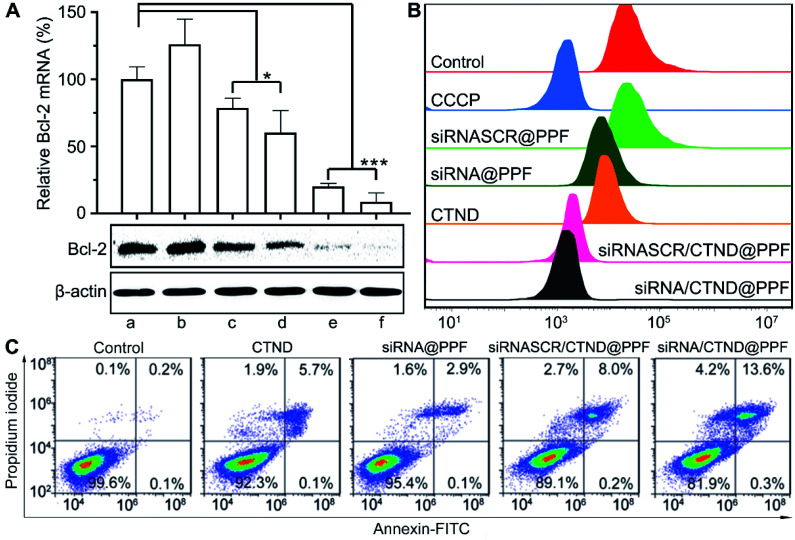
(A) Levels of Bcl-2 mRNA in B16F10 cells determined by quantitative real-time PCR and the expression of Bcl-2 protein determined by Western blot, *p < 0.05, ***p < 0.001, (a) control, (b) siRNASCR@PPF, (c) CTND, (d) siRNASCR/CTND@PPF, (e) siRNA@PPF, and (f) siRNA/CTND@PPF; (B) impacts of different compounds or composites on the red fluorescence intensity (*λ*_em_ = 488 nm) of TMRM after incubation with B16F10 cells for 24 h determined by flow cytometry; (C) apoptosis analysis of B16F10 cells after treatment with different samples for 24 h.

The silencing of the antiapoptotic Bcl-2 gene in B16F10 cells by siRNA/CTND@PPF was evaluated at the mRNA and protein levels by the real-time PCR and Western blotting assay, respectively. As shown in Fig. 5A, siRNASCR@PPF (b), CTND (c), and siRNASCR/CTND@PPF (d) had mild or moderate effects on the level of Bcl-2 mRNA and the expression of Bcl-2 protein as compared with the control (a). However, when the cells were incubated with Bcl-2 siRNA@PPF (e) or siRNA/CTND@PPF (f), the level of Bcl-2 mRNA sharply decreased (5.0 or 6.5 times), and the expression of Bcl-2 protein was also down-regulated. These data strongly suggest that the siRNA@PPF or siRNA/CTND@PPF NPs could transfer Bcl-2 siRNA effectively to the cells and lead to the downregulation of Bcl-2 protein.

It has been documented that inhibition of Bcl-2 is related to the dissipation of the mitochondrial inner transmembrane potential (Δ*ψ*_m_) before, during, or after mitochondrial outer membrane permeabilization (MOMP).^4,18^ Δ*ψ*_m_ could be measured by flow cytometry using a tetramethylrhodamine methyl ester (TMRM) fluorescent probe. As shown in Fig. 5B, compared to the control, siRNASCR@PPF barely changed the fluorescence; siRNA@PPF and CTND moderately decreased the fluorescence; siRNASCR/CTND@PPF and siRNA/CTND@PPF significantly decreased the fluorescence, comparable to the effect of the positive reference carbonyl cyanide *m*-chlorophenyl hydrazine (CCCP). The results suggest that both CTND and Bcl-2 siRNA can dissipate Δ*ψ*_m,_ and co-loading them in siRNA/CTND@PPF can aggravate the mitochondrial damage elicited by CTND, which may promote apoptosis.

To assess the apoptosis-inducing capability of siRNA/CTND@PPF due to the downregulation of Bcl-2 protein, B16F10 cells were subjected to an apoptosis analysis by staining with annexin V-FITC and propidium iodide. Compared to CTND, siRNASCR/CTND@PPF induced an increase in the total apoptosis rate (8.2% *vs.* 5.8%) due to the improved cellular uptake of CTDN by the NPs (Fig. 5C). A more significant increase in apoptosis was observed for siRNA/CTND@PPF, with a total apoptosis rate of 13.9% *vs.* 3.0% in the cells treated with siRNA@PPF, demonstrating that the downregulation of Bcl-2 protein by siRNA promoted the apoptosis of B16F10 cells, thus potentiating the antiproliferative activity of CTND.

### Cellular autophagy

**Fig. 6:**
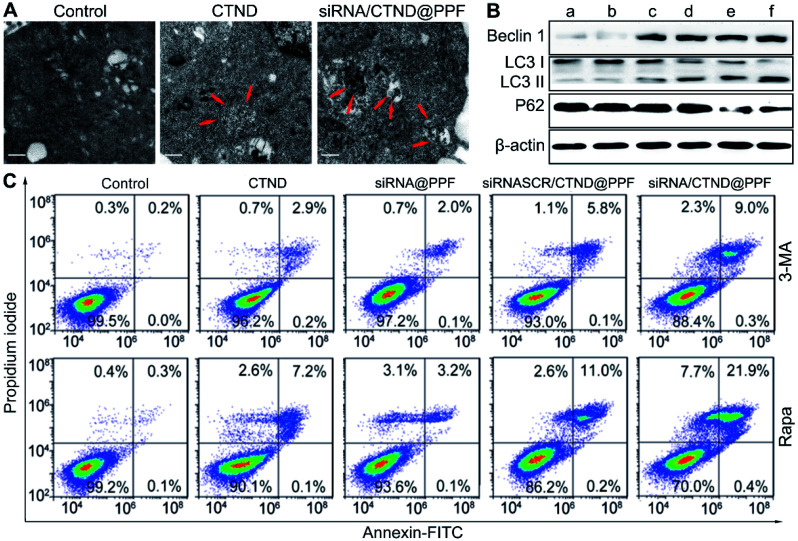
Enhancement of autophagy-associated apoptosis. (A) TEM images of untreated B16F10 cells and those treated with CTND and siRNA/CTND@PPF, respectively, showing autophagosomes with double-membrane structures (red arrow), scale bar = 0.5 μm; (B) expression of Beclin 1, LC3 and P62 proteins determined by Western blot analysis, (a) control, (b) siRNASCR@PPF, (c) CTND, (d) siRNASCR/CTND@PPF, (e) siRNA@PPF, and (f) siRNA/CTND@PPF; (C) apoptosis analysis of B16F10 cells after treatment with different samples in the presence of autophagy inhibitor 3-MA (10 mM) or inducer Rapa (100 nM).

Changes in Δ*ψ*_m_ are often accompanied by the regulation of the mitochondrial morphology.^19^ The morphological characteristics of B16F10 cells were further observed by TEM. As shown in Fig. 6A, siRNA/CTND@PPF induced the sequestration of the cytoplasmic material in autophagic vacuoles.^20^ Furthermore, the expression of autophagy-related proteins Beclin1, LC3 and P62 was determined. As shown in Fig. 6B, siRNA/CTND@PPF (f) upregulated the expression of the pro-autophagic markers Beclin1 and LC3 II and downregulated the expression of P62 more efficiently than CTND (c) or siRNA@PPF (e), implying that the combination of Bcl-2 siRNA and CTND can amplify the autophagy. To verify the relevance of the autophagy to apoptosis, B16F10 cells were treated with the autophagy inhibitor 3-methyladenine (3-MA) and the autophagy inducer rapamycin (Rapa), respectively. As shown in Fig. 6C, the apoptosis induced by siRNA/CTND@PPF decreased to 9.3% under the treatment of 10 mM 3-MA, but increased to 22.3% under the treatment of 100 nM Rapa. The results indicate that the downregulation of Bcl-2 protein activated the proapoptotic activity of CTND through initiating the autophagy. A recent report revealed that the activation of autophagy is critical for cell death through apoptosis and other signaling pathways.^21^ Previously we showed that the cytotoxicity of CTB resulted from multiple mechanisms of action; as an analogue of CTB, CTND was expected to act similarly. Unexpectedly, here we revealed that autophagy was also involved in the apoptotic process. Such a multispecific mechanism that targets several cell death pathways would have advantages for overcoming drug resistance and synergistically promoting apoptosis.^22^ As the development of anticancer copper complexes is still at its infant stage,^3^ the mechanism of action is in constant evolution over the years.

### Inhibition of tumor growth

**Fig. 7:**
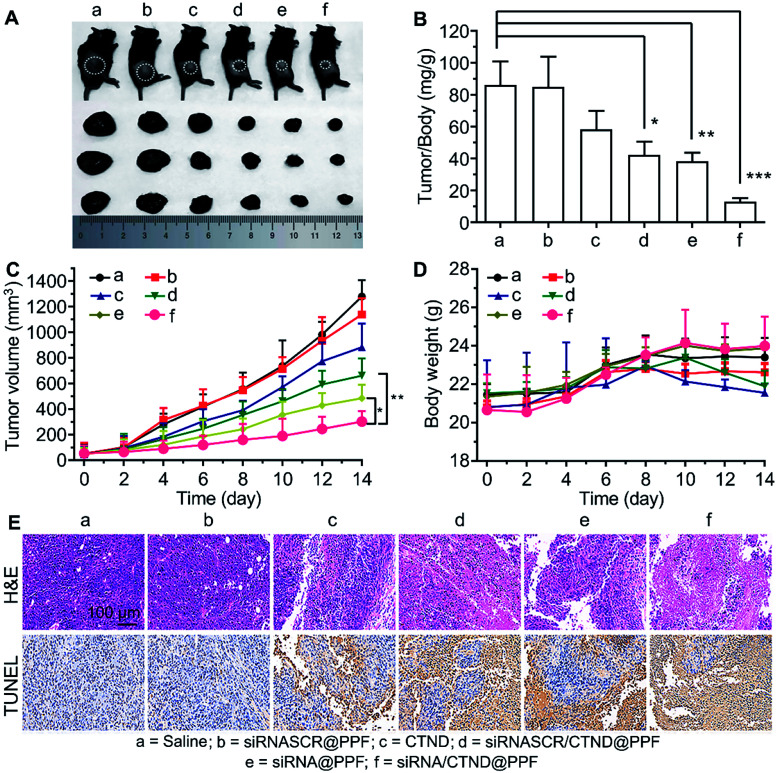
*In vivo* suppression of B16F10 tumor xenograft in C57BL/6 male mice in 14 days. (A) Representative tumor samples collected from the mice at the circled sites after intravenous injection of different compounds or composites; (B) tumor/body weight ratios in mice after the last determination (mean ± SD, *n* = 5), *p < 0.05, **p < 0.01, ***p < 0.001; (C) tumor growth curves after treatment with different samples, *p < 0.05, **p < 0.01; (D) body weight variations during the treatment; (E) H&E and TUNEL analyses of tumor tissues after treatment with different samples. Dosage: CTND = 5 mg kg^−1^ d^−1^, siRNA = 1.5 mg kg^−1^ every second day.

The *in vivo* antitumor efficacy of the NPs was investigated in C57BL/6 male mice bearing B16F10 tumor xenograft. As shown in Fig. 7A–C, the tumor growth was significantly suppressed after successive intravenous administration of siRNA/CTND@PPF, and the tumor resistance to CTND was largely overcome. Obviously, the efficacy of siRNA/CTND@PPF (f) was superior to that of CTND (c) or siRNA@PPF (e), suggesting that Bcl-2 siRNA can retrieve the activity of CTDN by interfering with the drug resistance mechanism. The body weight of the mice treated with siRNA/CTND@PPF did not change significantly as compared to that of the saline group, implying that the systemic toxicity of siRNA/CTND@PPF is much less than CTND (Fig. 7D). Moreover, after the treatment with siRNA/CTND@PPF, pathological images obtained using hematoxylin and eosin (H&E) staining displayed conspicuous cancer cell remission (Fig. 7E) with no obvious tissue abnormalities in the major organs (Fig. S13†). Images obtained using *in situ* terminal deoxynucleotidyltransferase-mediated UTP end labeling (TUNEL) assay showed that siRNA/CTND@PPF induced the most radical apoptosis in the tumor among the tested samples (Fig. 7E). These results indicate that siRNA/CTND@PPF effectively inhibited cell proliferation and increased apoptosis. No significant differences in the serum biochemical or routine blood indices were found in the mice receiving saline or NPs containing Bcl-2 siRNA and CTND (Fig. S14†), indicating that the systemic toxicity of the NPs was negligible.

## Conclusion

**Fig. 8:**
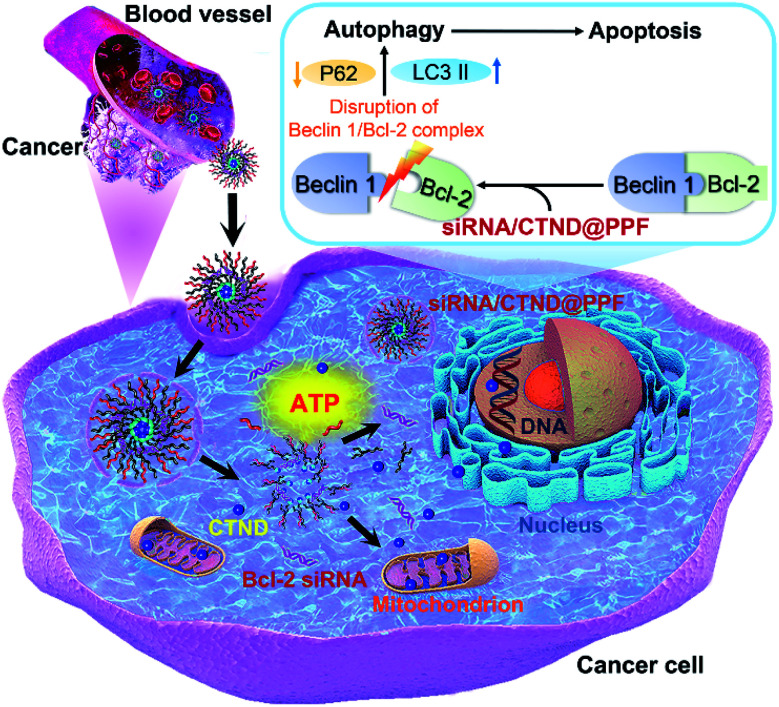
Schematic illustration of Bcl-2 siRNA/CTND@PPF mediated autophagic apoptosis.

Drug resistance is a common and complicated phenomenon for anticancer metallodrugs. Copper complexes are hopeful potential anticancer drugs; however, they also frequently encounter drug resistance. It has been shown that co-delivery of siRNA and cisplatin prodrug by NPs could enhance the tumor cell response to the drug.^9^ Here we demonstrated that a similar strategy could sensitize tumor cells to a seemingly inactive copper complex CTND. In order to deliver Bcl-2 siRNA and CTND safely and simultaneously to the tumor cells and achieve a synergistic antitumor effect, CTND was linked to the carrier polymer PPF through an ATP-responsive boronate ester and then assembled with Bcl-2 siRNA to form the siRNA/CTND@PPF NP. At cancer sites, siRNA/CTND@PPF was endocytosed into cancer cells and escaped from the endo-lysosomes because of the “proton sponge effect” of PEI.^17^ Once the NP encountered the cytoplasm, the elevated ATP would break the boronate ester bonds and lead to the release of CTND and Bcl-2 siRNA. CTND then targeted the mitochondria and cut mitochondrial DNA, as well as cleaved nuclear DNA.^4^ Meanwhile, Bcl-2 siRNA silenced the antiapoptotic Bcl-2 gene, broke the Beclin 1/Bcl-2 complex, modulated the expression of autophagy-related proteins LC3 and P62, induced autophagy and ultimately led to autophagic apoptosis (Fig. 8).

In recent years, multiple cocktail therapies have been developed to improve drug efficacy and reduce drug resistance, among them the combination of chemotherapeutics and siRNA is regarded as one of the promising schemes.^23^ The Bcl-2 protein family is closely related to the occurrence of tumor resistance to chemotherapeutics and is also involved in the mechanism of many apoptotic inducers. Accumulating evidence indicates that the high expression of Bcl-2 pro-survival protein positively correlates with the drug resistance;^2^ however, the effect of Bcl-2 siRNA on intrinsic tumor resistance to metallodrugs is rarely reported. In this study, we found that Bcl-2 siRNA could silence the Bcl-2 gene in B16F10 cells and curb intrinsic tumor resistance to CTND. Since CTND is a model complex representing a series of similar tumor-insensitive compounds, it is reasonable to believe that this methodology could be expanded to eliminate Bcl-2-induced resistance to other metal complexes. In other words, the strategy is relevant to no specific metal complex but to the expression of Bcl-2 protein. In principle, as long as the drug resistance towards a metal complex is induced by the Bcl-2 gene, this method is applicable. Generally, to adopt this strategy, three steps are required: (1) testing the sensitivity of cancer cells to a specific metal complex; (2) checking the expression of Bcl-2 protein in cancer cells; and (3) fabricating a Bcl-2-siRNA/metal complex co-delivery nanocarrier. In short, we showed a concrete example of how to circumvent the intrinsic tumor resistance to a copper complex in this study; by extension, silencing the Bcl-2 gene provides a general scheme for getting rid of the Bcl-2-related tumor resistance to metal-based anticancer drugs. Although the detailed anticancer mechanism of this nanocomposite still needs further elucidation, the basic conception of the design applies to other metallodrugs.

## Conflicts of interest

The authors declare no conflict of interest.

## Supplementary Material

SC-012-D0SC06680J-s001
