# Supplementary material for: Surmounting tumor resistance to metallodrugs by co-loading a metal complex and siRNA in nanoparticles
Source: Chem Sci. 2021 Feb 9;12(12):4547–56. doi: 10.1039/d0sc06680j (PMC8179575; doi:10.1039/d0sc06680j)
Supplement: SC-012-D0SC06680J-s001 [file SC-012-D0SC06680J-s001.pdf]

Electronic Supplementary Material (ESI) for Chemical Science.
This journal is © The Royal Society of Chemistry 2021

Electronic Supplementary Information (ESI)

Surmounting Tumor Resistance to Metallodrugs by Co-loading Metal Complex and siRNA in Nanoparticles

Hongzhi Qiao*, Lei Zhang, Dong Fang, Zhenzhu Zhu, Weijiang He, Lihong Hu, Liuqing Di, Zijian Guo, Xiaoyong Wang*

1. Supplementary figures and tables

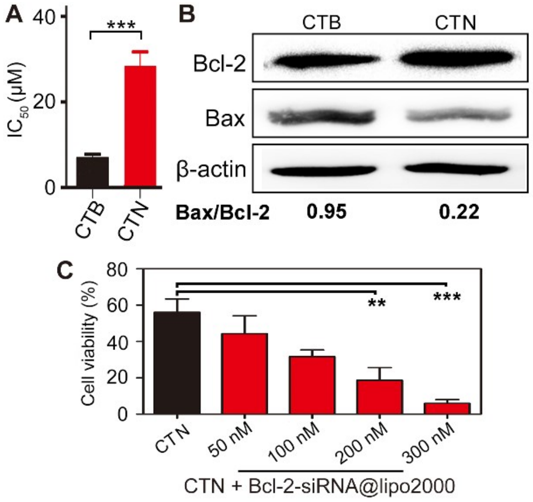

Fig. S1 A) Cytotoxicity of CTB and CTN after incubation with B16F10 cells for 24 h, measured by the MTT assay; B) expression of Bcl-2 and Bax in B16F10 cells analysed by Western blot; and C) viability of B16F10 cells after incubation with CTN or CTN plus Bcl-2-siRNA@lipo2000 determined by the MTT assay. **p<0.01, ***p<0.001.

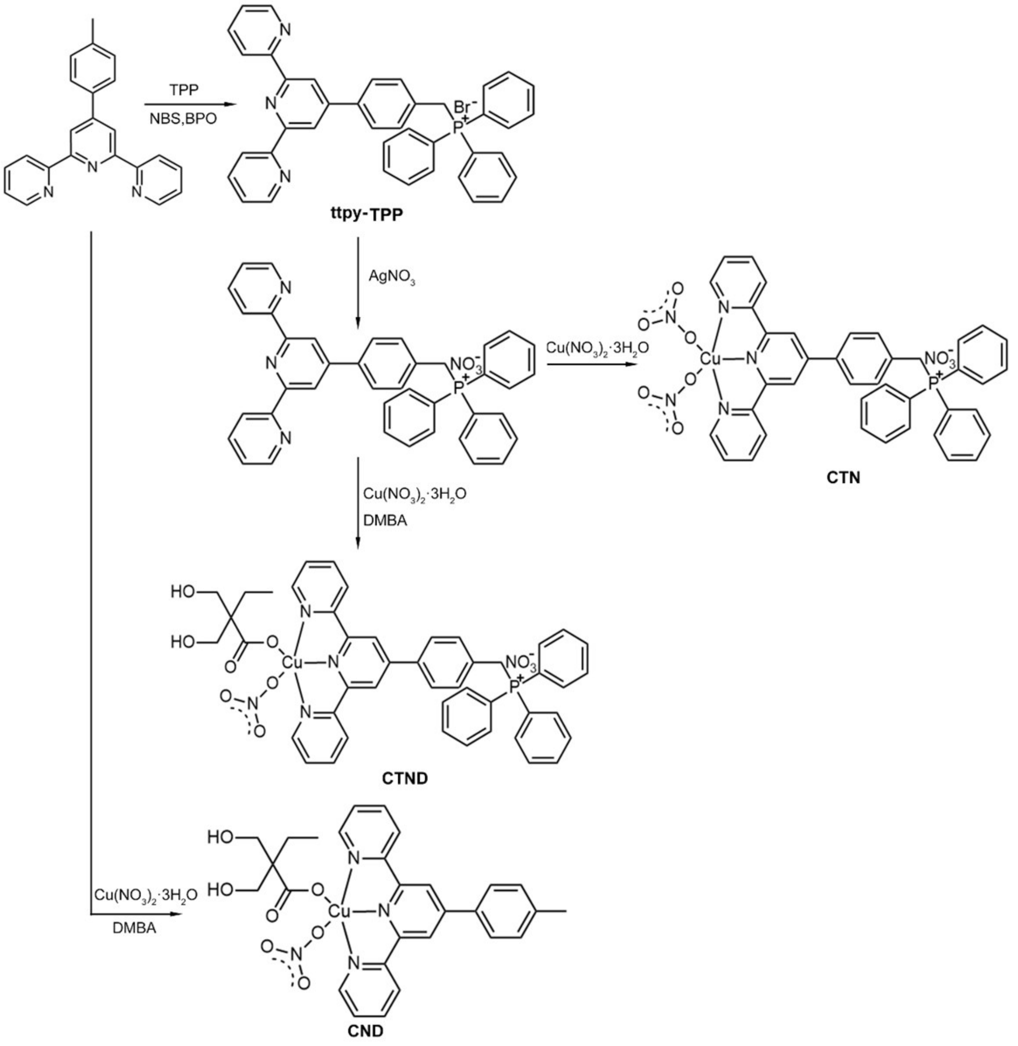

Scheme S1. Synthetic routes to CTN, CTND, and CND.

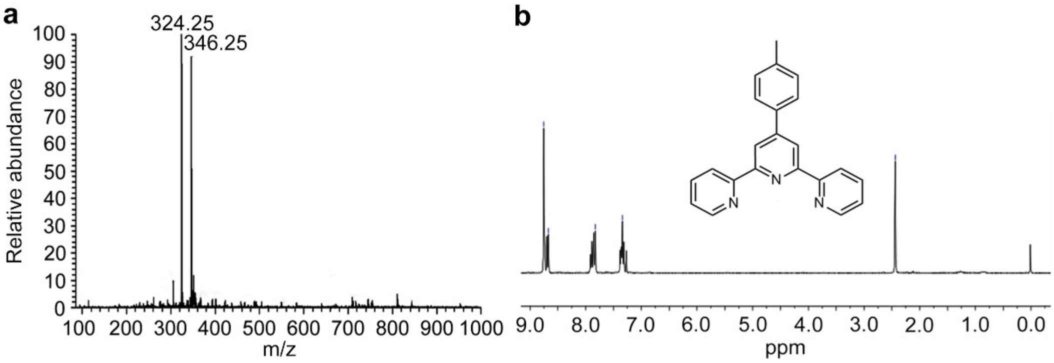

Fig. S2 a) ESI-MS spectrum of ttpy in methanol; b) ^{1}H NMR spectrum of ttpy in DMSO-d_6.

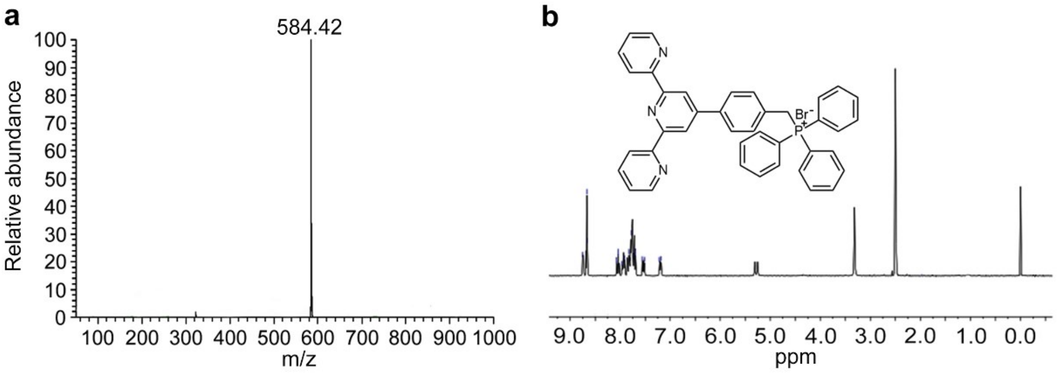

Fig. S3 a) ESI-MS spectrum of ttpy-TPP in methanol; b) ^{1}H NMR spectrum of ttpy-TPP in DMSO-d_6.

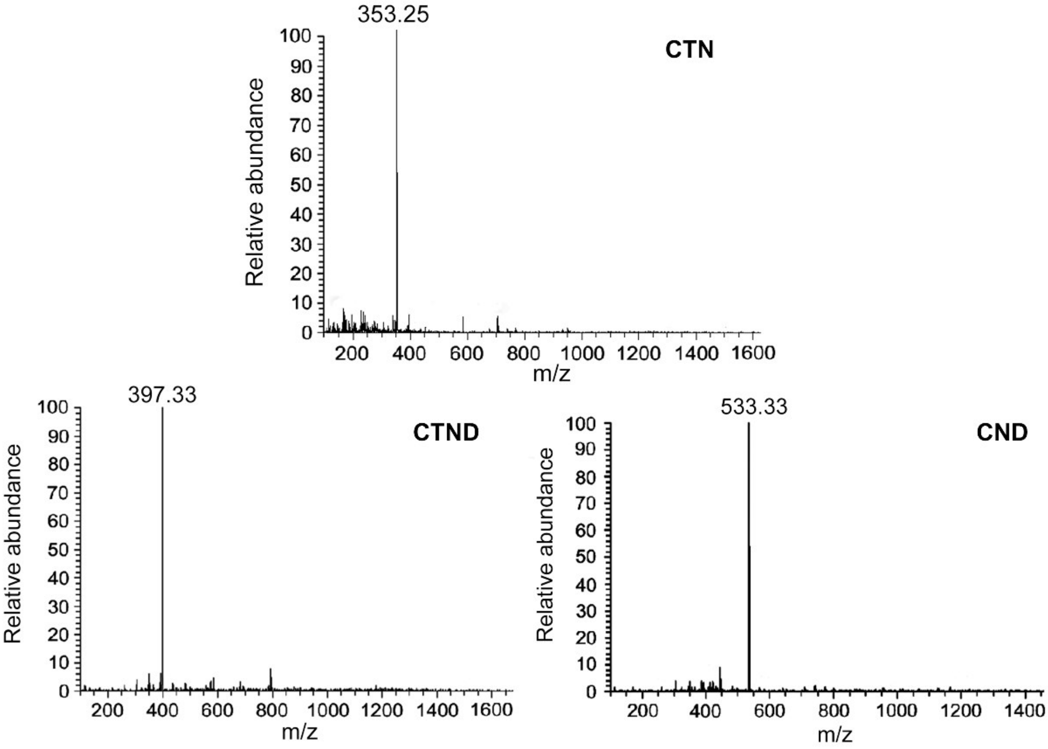

Fig. S4 ESI-MS spectra of CTN, CTND, and CND in methanol.

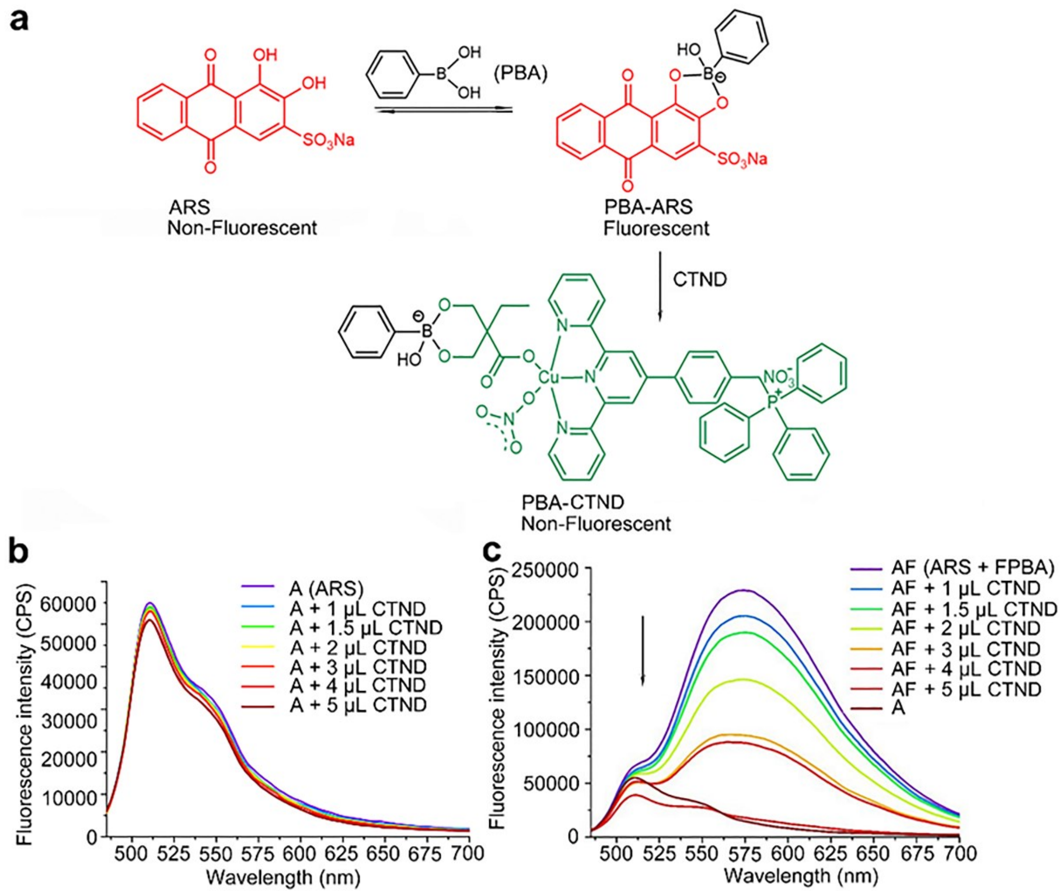

Fig. S5 a) Competitive binding of the ternary system consisting of ARS, PBA and CTND; fluorescence spectra of ARS (b) and ARS-FPBA; and (c) solutions with increasing concentrations of CTND after 5 min of incubation in HEPES buffer (HB, 10 mM, pH 7.4).

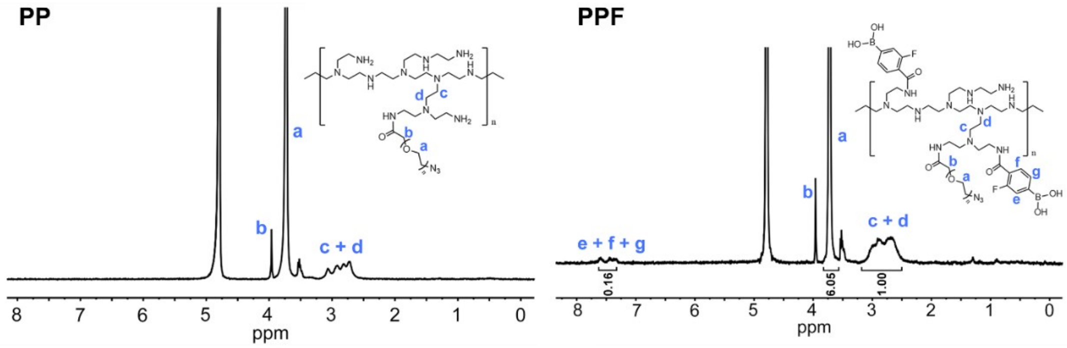

Fig. S6 ^{1}H NMR spectra of PP and PPF in D_2O.

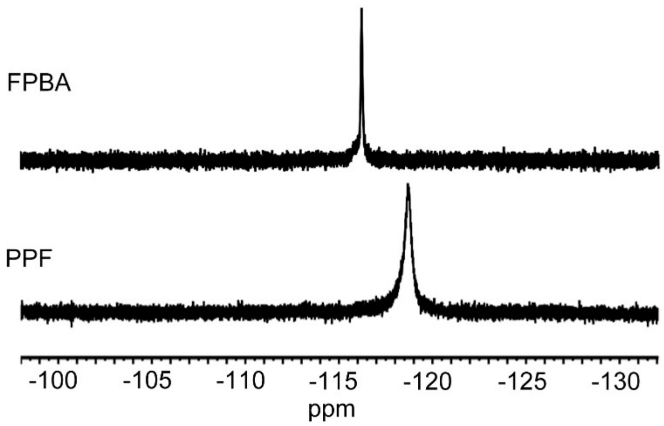

Fig. S7 ^{19}F NMR spectra of FPBA and PPF in D_2O.

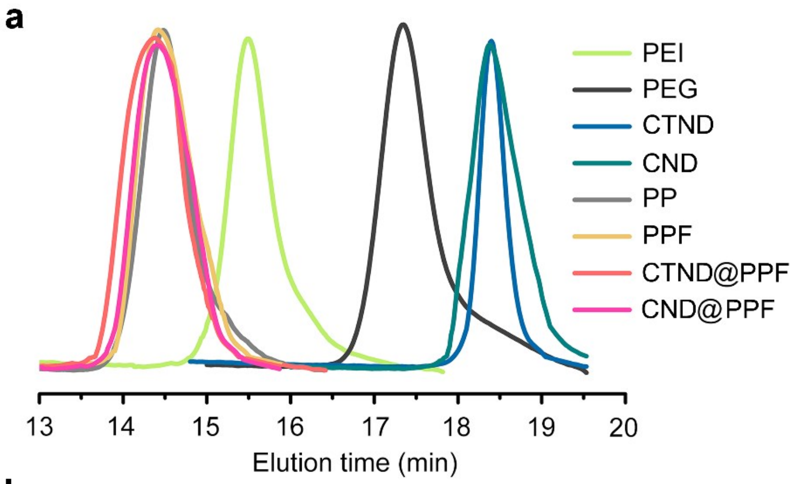

b

**Polymer**	M_n	M_w/M_n
PEI	9986	1.09
CTND@PPF	42291	1.28
CND@PPF	42085	1.25

Fig. S8 a) GPC traces of PEI, PEG, CTND, CND, PP, PPF, CTND@PPF, and CND@PPF, with 0.2 M $NaNO_3$ as the eluent at a flow rate of 1 mL min^{-1}; b) number-average molecular weight (M_n) and polydispersity index (M_w/M_n) of PEI, CTND@PPF, and CND@PPF.

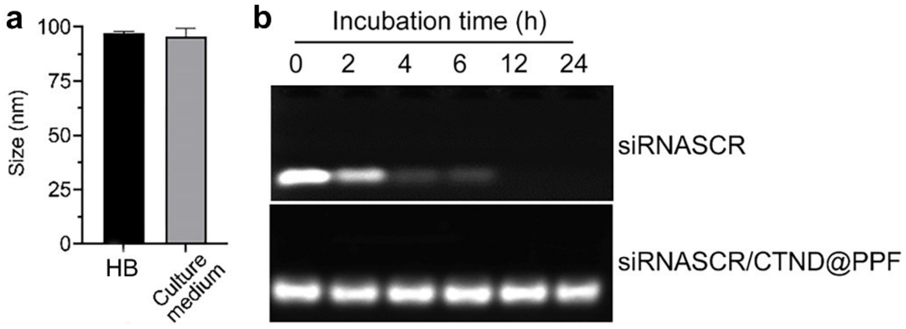

Fig. S9 a) Stability of siRNASCR/CTND@PPF in HB (10 mM, pH 7.4) or culture medium measured by a dynamic light scattering (DLS) analyzer after keeping at room temperature for 48 h; and b) stability of naked siRNASCR and siRNASCR/CTND@PPF in fetal bovine serum examined by analysing agarose gel electrophoresis of siRNASCR-loaded complexes upon treatment with FBS, with FBS-treated siRNASCR as the positive control.

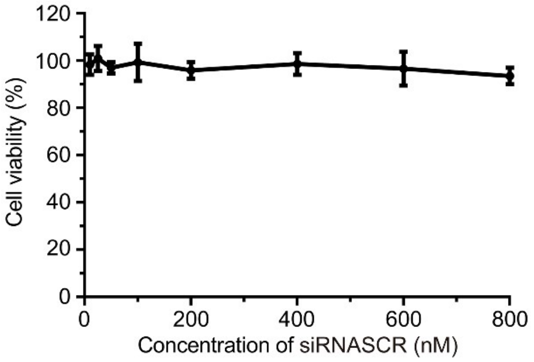

Fig. S10 *In vitro* cytotoxicity of siRNASCR@PPF against B16F10 cells.

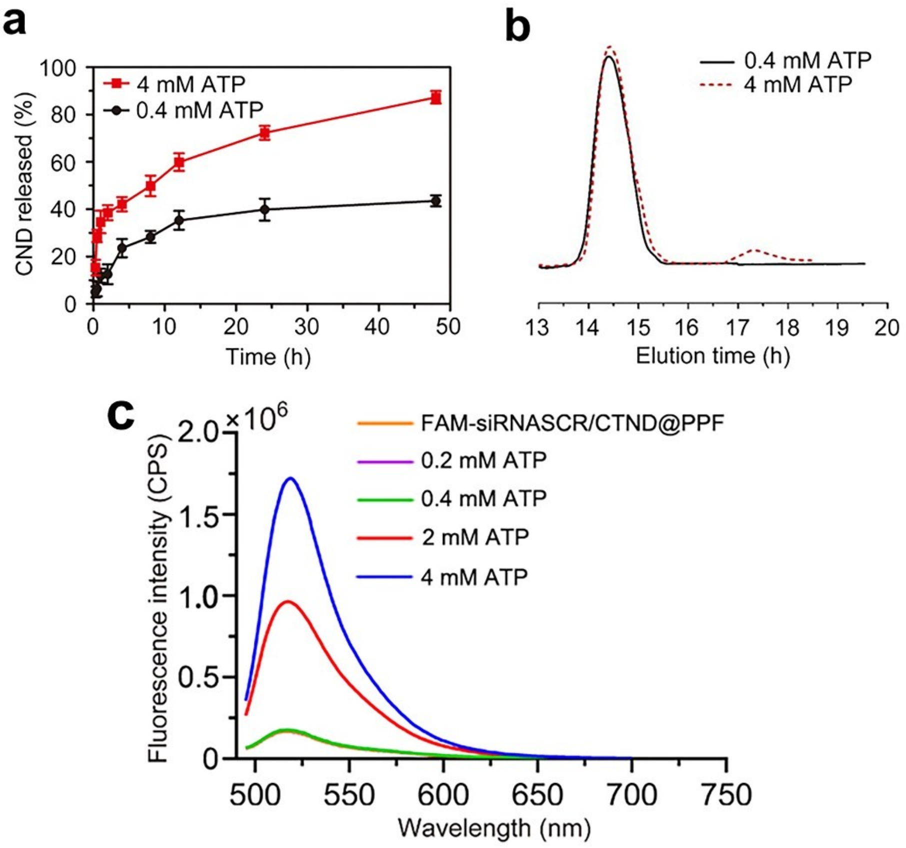

Fig. S11 a) *In vitro* CND release from siRNASCR/CND@PPF at different ATP concentrations (mean ± SD, n = 3); b) GPC curve of CND@PPF at different ATP concentrations after 4 h incubation; and c) fluorescence spectra of FAM-siRNASCR released from FAM-siRNASCR/CTND@PPF at different ATP concentrations after incubation for 2 h.

Table S1. ATP-triggered release of CTND from siRNASCR/CTND@PPF in B16F10 cells in terms of Cu content determined by ICP-MS (mean ± SD, n = 3).

	Mito (ng/mg)	Nucleus (ng/mg)
37 °C 0 h	36.1 ± 1.0	99.1 ± 2.7
37 °C 4 h	49.0 ± 1.0	129.1 ± 3.1
37 °C 6 h	57.3 ± 2.0	145.4 ± 3.0
37 °C IAA 4 h	24.4 ± 1.8	87.7 ± 1.7
37 °C IAA 6 h	18.9 ± 2.4	69.2 ± 2.1
4 °C 4 h	11.2 ± 1.3	57.7 ± 2.8
4 °C 6 h	5.7 ± 0.9	46.0 ± 1.3

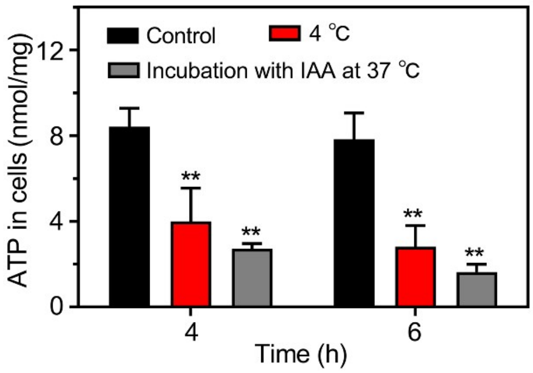

Fig. S12 ATP content in B16F10 cells after different treatments. **$p < 0.01$.

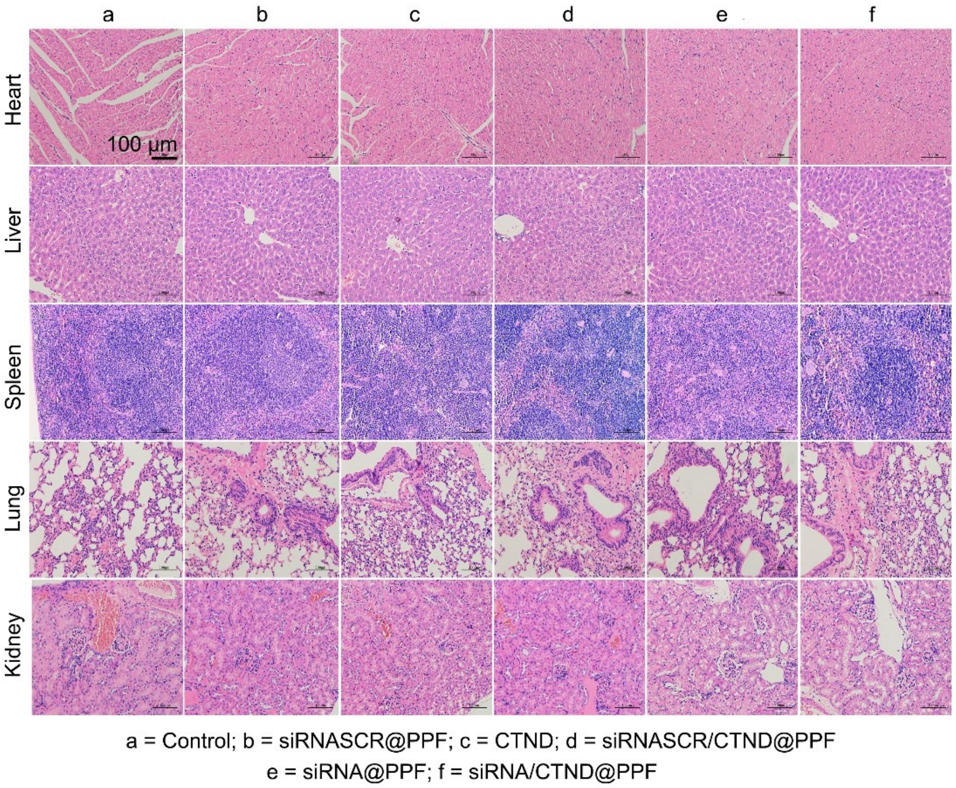

Fig. S13 H&E analysis of major organs after treatment with saline, siRNASCR@PPF, CTND, siRNASCR/CTND@PPF, siRNA@PPF, and siRNA/CTND@PPF, respectively. Scale bar = 100 µm.

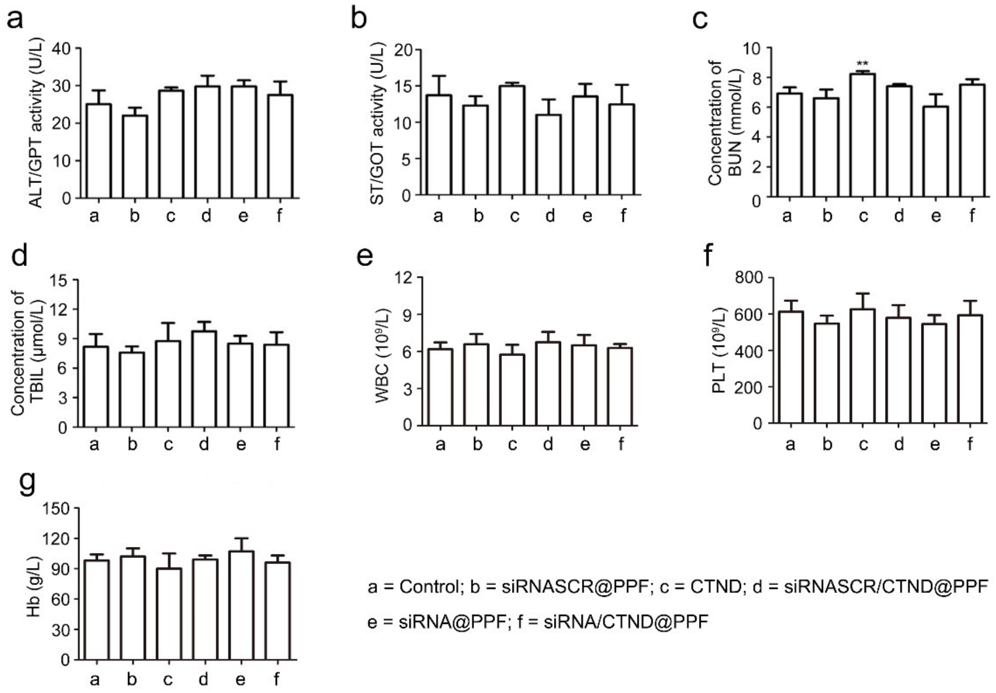

Fig. S14 Serum biochemical indices and routine blood indexes in mice treated with different compounds or composites (mean ± SD, n = 5). $**p < 0.01$ compared to saline.

2. Experimental procedures

Chemicals and reagents. 2-Acetylpyridine, p-tolualdehyde, dimethylolbutyric acid (DMBA) and triphenylphosphine (TPP, 98.0%) were purchased from Energy Chemical Co., Ltd. (Shanghai, China). N-bromosuccinimide (NBS, 99.0%), benzoyl peroxide (BPO, 98.0%), ammonium acetate (≥ 98.0%), copper(II) nitrate trihydrate ($Cu(NO_3)_2 \cdot 3H_2O$, 99.0~102.0%), and silver nitrate ($AgNO_3$, ≥ 99.8%) were obtained from Sinopharm Chemical Reagent Co., Ltd. (Beijing, China). Alizarin Red S (ARS), 3-fluoro-4-carboxyphenylboronic acid (FPBA, 98%) and branched polyethyleneimine (PEI, 10 kDa) were provided by Aladdin Biochemical Technology Co., Ltd. (Shanghai, China). Azide-PEG-carboxyl (N_3-PEG-COOH, 2 kDa) was obtained from Ponsure Biotechnology Co., Ltd. (Shanghai, China). 1-Ethyl-3(3-dimethylaminopropyl) carbodiimide (EDC), adenosine 5'-triphosphate disodium salt hydrate (ATP), iodoacetic acid (IAA), 3-methyladenine (3-MA) and rapamycin were purchased from Sigma-Aldrich Co., LLC (St. Louis, MO, USA). Scrambled siRNA (siRNASCR), FAM-labeled scrambled siRNA (FAM-siRNASCR) and siRNA targeting Bcl-2 mRNA (Bcl-2 siRNA) were synthesized by GenePharma Co., Ltd. (Shanghai, China). The sequences of siRNASCR and Bcl-2 siRNA are as follows: siRNASCR (sense strand) 5'-UUCUCCGAACGUGUCACGUTT-3', (antisense strand) 5'-ACGUGACACGUUCGGAGAATT-3'; Bcl-2 siRNA (sense) 5'-GUACAUCCAUUAUAAGCUGUCTT-3', (antisense strand) 5'-GACAGCUUAUAAUGGAUGUACTT-3'.

Synthesis of PP. Polyethylene glycol (N_3-PEG) containing carboxyl groups was conjugated to the primary amino groups of PEI via a standard EDC chemistry. Specifically, N_3-PEG-COOH (0.60 g, 0.3 mmol carboxyl) and EDC (0.12 g, 0.60 mmol) were dissolved in deionized water and stirred for 30 min. PEI (0.10 g, 0.6 mmol primary amine groups) were added dropwise into the solution. The mixture was kept at room temperature in a nitrogen atmosphere for 12 h; afterwards, the mixture was dialyzed against deionized water for 24 h. The dialyzed solution was freeze-dried to obtain PP (Yield: 95%).

Synthesis of PPF. The polymeric carrier PPF was synthesized via the standard EDC conjugation method. FPBA (0.055 g, 0.30 mmol) and EDC (0.12 g, 0.60 mmol) were dissolved in methanol/deionized water (1/1, v/v) and stirred for 30 min, and then PP (0.20 mmol primary amine groups) dissolved in methanol/deionized water (1/1, v/v) was added dropwise into the solution. After 12 h in N_2 atmosphere, the reaction solution was dialyzed against deionized water for 24 h. Finally, the dialyzed solution was lyophilized to obtain PPF (Yield: 93%). ^1H- and ^{19}F NMR spectra were obtained from a Bruker 400 MHz spectrometer. Molecular weight of the sample was recorded by a gel permeation chromatography system (PL-GPC 50; Agilent Technologies, USA) at 35 °C with PEG/PEO as the standard for calibration. Deionized water containing $NaNO_3$ (0.2 M) at a flow rate of 1.0 mL min^{-1} was used as the mobile phase.

Synthesis of CTN. CTB and ligand ttpy-TPP were obtained using a previously reported method.[1] CTN was synthesized as follows: ttpy-TPP was dissolved in water/ethanol (1/3, v/v), and excess silver nitrate was added under stirring until the reaction finished. The sediment was filtered, and the filtrate was dried. The obtained white solid was washed with deionized water three times and dried in vacuo. CTN was obtained by mixing a methanol solution of $Cu(NO_3)_2 \cdot 3H_2O$ (1.13 mmol) and silver nitrate-treated ttpy-TPP (0.75 mmol). The mixture was heated to reflux for 4 h at 40 °C.

Methanol was removed, and the resulting green solid was washed with acetone and diethyl ether. The product was dried in vacuo until the weight remain constant (Yield: 69%). ttpy-TPP: ^{1}H NMR (300 MHz, DMSO-*d6*): δ = 8.75 (m, 2H), 8.68 (m, 1H), 8.66 (m, 3H), 8.04 (m, 2H), 7.93 (m, 3H), 7.85 (d, *J* = 7.8Hz, 2H), 7.76 (m, 12H), 7.53 (m, 2H), 7.20 (dd, *J* = 2.3Hz, 2H), 5.27 (d, *J* = 15.9Hz, 2H). Electrospray ionization mass spectrometry (ESI-MS) spectra were obtained using an LCQ fleet ESI-MS spectrometer in positive mode (Thermo Scientific, USA). LRMS (ESI, methanol) *m/z*: $[M – Br]^+$ calcd for $C_{40}H_{31}N_3P$, 584.23; found, 584.42; CTN: LRMS (ESI, methanol) *m/z*: $[M – 2NO_3]^{2+}$ calcd for $C_{40}H_{31}N_4O_3CuP$, 708.13/2; found, 353.25.

Synthesis of CTND. ttpy-TPP was dissolved in water/ethanol (1/3, v/v), and excess silver nitrate was added under stirring until the reaction was completed. The sediment was removed by filtration, and the filtrate was dried. CTND was obtained by mixing methanol solution of DMBA (5.56 mmol) and $Cu(NO_3)_2·3H_2O$ (1.13 mmol). After stirring for 1 h, a methanol solution of silver nitrate-treated ttpy-TPP (0.75 mmol) was added to the above solution, and the mixture was heated to reflux for another 4 h at 40 °C. Methanol was removed and the resulting green solid was washed with acetone and diethyl ether. The solid was dried in vacuo until the weight was constant (Yield: 64%). CTND: LRMS (ESI, methanol) *m/z*: $[M – 2NO_3]^{2+}$ calcd for $C_{46}H_{42}CuN_3O_4P$, 794.22/2; found, 397.33.

Synthesis of CND. Ligand ttpy was obtained according to a reported method.[2] CND was obtained by mixing methanol solution of DMBA (5.6 mmol) and $Cu(NO_3)_2·3H_2O$ (1.1 mmol). After stirring for 1 h, methanol solution of ttpy (0.75 mmol) was added and heated to reflux for another 4 h at 40 °C. Methanol was removed and the resulting green solid was washed with acetone and diethyl ether. The product was dried in vacuo until the weight kept constant (Yield: 67%). ttpy: ^{1}H NMR (300 MHz, DMSO-d_6): δ = 8.75 (s, 3H), 8.74 (m, 1H), 8.68 (m, 2H), 7.88 (m, 3H), 7.83 (s, 1H), 7.36 (m, 3H), 7.31 (s, 1H), 2.44 (s, 3H). LRMS (ESI, methanol) *m/z*: $[M + H]^+$ calcd for $C_{22}H_{18}N_3$, 324.15; found, 324.25. *m/z*: $[M + Na]^+$ calcd for $C_{22}H_{17}N_3Na$, 346.13; found, 346.25; CND: LRMS (ESI, methanol) *m/z*: $[M – NO_3]^+$ calcd for $C_{28}H_{28}CuN_3O_4$, 533.14; found, 533.33.

Synthesis of CTND@PPF and CND@PPF. PPF (50 mg) was dissolved in anhydrous THF, and a solution of THF containing 1.5 equivalent of CTND or CND was added dropwise (per 4-carboxy-3-fluorophenylboronic acid). The reaction mixture was stirred for 12 h at room temperature in N_2 atmosphere; afterwards, the mixture was dialyzed against methanol for 24 h. The polymer was concentrated by evaporation, producing a green viscous oil. A green solid was obtained after resting for 1 h. Drug-loading efficiency DLE (%) = (weight of loaded CTND/total weight of NPs) × 100%.

Preparation and characterization of siRNASCR/CTND@PPF and siRNASCR/CND@PPF. siRNASCR (20 μM, 1 μL) and CTND@PPF or CND@PPF (9 μL) dissolved in HEPES buffer (HB, 10 mM, pH 7.4) were mixed at different N/P (1, 3, 5, 7 and 9) ratios to obtain siRNASCR/CTND@PPF or siRNASCR/CND@PPF. The mixture was kept at room temperature for 30 min after gentle vortexing and then applied to a 1% agarose gel electrophoresis with EB (0.5 mg mL^{-1}) in tris-acetate buffer at 100 V using a Gel-DocTM XR imaging system (Bio-Rad, USA). siRNASCR@PPF were prepared by mixing siRNASCR (1 μL, 20 μM) and PPF (9 μL) in HB at different N/P (1, 2, 3, 4 and 5) ratios.

Cytotoxicity of CTB and CTN. B16F10 cells were cultured in Dulbecco's modified eagle medium (DMEM), which were supplemented with penicillin (100 U/ml), streptomycin (100 U/ml), and heat-

inactivated fetal bovine serum (10%) at 37 °C in a humidified atmosphere containing 5% CO_2 incubator. B16F10 cells (5 × 10^3 cells per well) were seeded in a 96-well plate and cultured overnight. The culture media were replaced by fresh media containing CTB (1–10 μM) or CTN (10–40 μM) and cultured for 24 h. The cells were subjected to the MTT assay after washed with PBS three times.

Competitive binding. Fluorescence intensity changes of ARS were monitored using a previously reported method.[3] ARS (9.0 × 10^{-6} M) and FPBA (2.0 × 10^{-3} M) were dissolved in HB (10 mM, pH 7.4), obtaining solution A. A specified amount of CTND was dissolved in solution A to make a final concentration of 9.0 × 10^{-6} M ARS, 2.0 × 10^{-3} M FPBA and 0.2 × 10^{-3} CTND in HB (solution B). A series of fixed volumes of solution B were mixed with solution A to get a constant concentration of ARS and FPBA and a gradient concentration of CTND. The fluorescence intensity of each sample was recorded at an excitation wavelength of 468 nm after 5 min.

Stability of siRNASCR/CTND@PPF and siRNA. siRNASCR (20 μM, 1 μL) and CTND@PPF (9 μL) were dissolved in HB (10 mM, pH 7.4) and mixed at N/P 5 to obtain siRNASCR/CTND@PPF. The mixture was kept at room temperature for 48 h. The particle size of siRNASCR/CTND@PPF in HB (10 mM, pH 7.4) or culture medium was measured by a dynamic light scattering (DLS) analyzer. To evaluate the stability of siRNA in 10% FBS, siRNASCR or siRNASCR/CTND@PPF (20 μM siRNASCR) at N/P 5 was incubated with 10% FBS (1:1, v/v) for different durations (0, 2, 4, 8, 12 and 24 h) at 37 °C. The samples were loaded to a 1% agarose gel with EB (0.5 mg/mL) in tris-acetate buffer at 100 V. Before gel electrophoresis, the sample solution was treated with 1 μL heparin sodium salt solution (2 mL:12500 IU) for 30 min to free siRNASCR from the NP.

ATP-dependent release of CTND and siRNASCR from siRNASCR/CTND@PPF. The release behavior of CTND or CND stimulated by different concentrations of ATP was detected by HPLC. The release of siRNASCR was monitored by fluorescence determination. Briefly, siRNASCR/CTND@PPF or siRNASCR/CND@PPF (N/P 5) dissolved in HB (10 mM, pH 7.4) was added to individual dialysis bag (MWCO 3 kDa) to dialyze against 50 mL HB (10 mM, pH 7.4) in two different ATP concentrations with a gentle shaking on a vibrating table at 37 °C. At set time points, 5 mL HB was collected, followed by replenishment of the same volume of fresh HB. The content of CTND and CND was measured by HPLC (Shimadzu LC-20A, Japan; 0.1% HNO_3/MeOH = 60/40, v/v; 0.8 mL min^{-1}).

To detect the release of siRNASCR, free FAM-siRNASCR and FAM-siRNASCR/CTND@PPF (2 μM FAM-siRNASCR, N/P 5) dissolved in HB was incubated at different ATP concentrations for 0, 2, 4, 6, 12 and 24 h, respectively, at 37 °C. Each sample was centrifuged at 14000 rpm for 10 min. Fluorescence intensity (E_x/E_m = 480/517 nm) of FAM-siRNASCR released in the supernatant was measured by a spectrofluorometer (PerkinElmer LS-50B, USA).

Intracellular ATP-dependent CTND release. B16F10 cells (1 × 10^5 cells per well) were seeded in 6-well plates and cultured overnight. siRNASCR/CTND@PPF (5 μM CTND; N/P 5) was added and incubated for 4 h. Medium was replaced by fresh serum-free DMEM and the cells was incubated for another 4 or 6 h. Afterwards, mitochondria and nuclei were separately isolated and digested. Cu content in mitochondria and nuclei was quantified by ICP-MS. To investigate the relationship between the release of CTND and ATP content, a low temperature (4 °C) and IAA (100 μM) was

utilized to inhibit the production of ATP. The ATP content produced in cells was measured using an ATPlite Assay Kit.

Cytotoxicity of siRNASCR@PPF. B16F10 cells (5×10^3 cells per well) were seeded in 96-well plates and cultured overnight. The medium was replaced with fresh one containing siRNASCR@PPF with concentrations of siRNASCR ranging from 10 to 800 nM. The cells were further incubated for 24 h after washed with PBS three times and then tested by the MTT assay.

Intracellular uptake. B16F10 cells (1×10^5 cells per well) were seeded in 6-well plates and cultured overnight. The medium was replaced with fresh one containing FAM-siRNASCR or FAM-siRNASCR/CTND@PPF and incubated for 1, 2, 4 and 6 h with 200 nM FAM-siRNASCR. At each time point, cells were trypsinized without EDTA, collected and subjected to flow cytometry analysis (BD Accuri C6, USA).

Mitochondrial uptake of Cu. B16F10 cells were cultured in 100 mm dishes and exposed to CTND, CTND@PPF, siRNASCR/CTND@PPF, CND, CND@PPF, and siRNASCR/CND@PPF (5 μM CTND or CND; N/P 5) for 12 h. The cells were collected and mitochondria were isolated by a mitochondria isolation kit. The mitochondria were digested by successive addition of 100 μL concentrated nitric acid, 50 μL hydrogen peroxide (30%) and 100 μL concentrated hydrochloric acid for 1 h at 95 °C. In addition, the cells under the same culture conditions were collected and digested. Cu content in mitochondria and whole cells was analyzed using ICP-MS (PerkinElmer ELAN9000, USA). The protein in each sample was extracted and measured using a BCA protein assay kit.

Intracellular tracking. B16F10 cells (1×10^5 cells per well) were seeded in glass-bottomed dishes (35 mm × 10 mm) and incubated overnight. The cells were then incubated with FAM-siRNASCR@PPF (N/P 3) and FAM-siRNASCR/CTND@PPF (200 nM FAM-siRNASCR; N/P 5) for 1 and 4 h, respectively. LysoTracker red was added into each dish and stayed for 30 min; DAPI was added and remained for 10 min followed by observation with CLSM (Leica TCS SP5, Germany). Fluorescence and tracking analysis were performed using ZEN 2009 Light Edition.

Intracellular Bcl-2 gene-silencing. B16F10 cells (1×10^5 cells per well) were seeded in 6-well plates and treated with siRNASCR@PPF, CTND, siRNASCR/CTND@PPF, siRNA@PPF, and siRNA/CTND@PPF, respectively, at an equivalent siRNASCR or siRNA dose of 200 nM for 24 h. Total cellular mRNA was extracted. The level of Bcl-2 mRNA was measured by qPCR, and the program was performed under heat-cycling conditions for 5 min at 95 °C, followed by 40 cycles for 30 s at 95 °C, 15 s at 58 °C, and 15 s at 68 °C. For Bcl-2, the sense and antisense primer sequences were 5'-AGGAGCAGGTGCCTACAAGA-3' and 5'-GCATTTTCCCACCACTGTCT-3', respectively. For β-actin, the sense and antisense primer sequences were 5'-TGTTACCAACTGGGACGACA-3' and 5'-AGGAGCAGGTGCCTACAAGA-3', respectively. B16F10 cells were washed with PBS three times and collected by trypsinization and centrifugation. The expression of Bcl-2 protein was analyzed by Western bolt.

Cell apoptosis assay. B16F10 cells (1×10^5 cells per well) were seeded in 6-well plates, cultured overnight, and transfected with siRNASCR@PPF (N/P 3), CTND, siRNASCR/CTND@PPF, siRNA@PPF (N/P 3), and siRNA/CTND@PPF (10 μM CTND; N/P 5) for 24 h. The cells were

then harvested by centrifugation, rinsed with cold PBS. Apoptosis was evaluated by flow cytometry using an annexin V-FITC/PI apoptosis analysis kit.

Mitochondrial membrane potential. B16F10 cells (1×10^5 cells per well) were seeded in 6-well plates and exposed to a series of siRNASCR@PPF, siRNA@PPF (N/P 3), CTND, siRNASCR/CTND@PPF, and siRNA/CTND@PPF (20 μM CTND; N/P 5), respectively, for 24 h. At predetermined time interval, cells were trypsinized, collected and incubated with 1 × TMRM dyeing buffer. Each sample was analyzed by flow cytometry. CCCP (10 mM) was used as a positive control.

Induction of autophagic apoptosis. B16F10 cells (1×10^5 cells per well) were seeded in 6-well plates and treated with siRNASCR@PPF, CTND, siRNASCR/CTND@PPF, siRNA@PPF, and siRNA/CTND@PPF, respectively, at the siRNASCR or Bcl-2 siRNA dose of 200 nM for 24 h. Detection of Beclin1, LC3 and P62 proteins were processed according to the Western blotting protocol described above.

B16F10 cells (1×10^5 cells per well) were seeded in 6-well plates. The cells were incubated with 100 nM of rapamycin for 2 h and then transfected with CTND, siRNASCR/CTND@PPF, siRNA@PPF (N/P 3), and siRNA/CTND@PPF (10 μM CTND ; N/P 5), respectively, containing 100 nM rapamycin or 10 mM 3-MA for 24 h. All cells were harvested and evaluated by flow cytometry using an annexin V-FITC/PI apoptosis analysis kit.

***In vivo* antitumor activity and safety evaluation.** C57BL/6 male mice (20 ± 2 g) were fed with a standard laboratory diet. Animal study protocol was approved by the Institutional Animal Care and Use Committee of Nanjing University, and all animal experiments were carried out according to the approved guidelines. B16F10 tumor-bearing mice were weighed and divided into 7 groups with 5 mice per group. When the tumor volume reached ca 50 mm^3, the mice were administered intravenously via the tail vein with 200 μL siRNASCR@PPF, siRNA@PPF, CTND, siRNASCR/CTND@PPF, and siRNA/CTND@PPF, respectively, at an equivalent siRNASCR or Bcl-2 siRNA dose of 1.5 mg Kg^{-1} on every second day for 14 days. Control group was given 0.9% normal saline. Tumor size and body weight were recorded during the treatment, and tumor volume was calculated on $V = 0.5 \times L \times W^2$, where L and W represent the length and width of the tumor, respectively. At the end of experiments, the animals were sacrificed, and main organs (heart, liver, spleen, lung, and kidney) and tumor tissue were collected and prepared for H&E staining. Tumor sections were stained with TUNEL for apoptosis examination. Biochemical parameters and blood routine indices of blood samples were quantified using corresponding test kits.

Statistical analysis. Statistical analysis of data was performed using one-way ANOVA (SPSS software). Results were presented as mean ± SD, and $p < 0.05$ indicated statistically significant difference.

References

[1] W. Zhou, X. Y. Wang, M. Hu, C. C. Zhu, Z. J. Guo, *Chem Sci*. **2014**, 5, 2761-2770.

[2] C. Bhaumik, S. Das, D. Saha, S. Dutta and S. Baitalik, *Inorg. Chem.* **2010**, 49, 5049-5062.

[3] W. Scarano, H. T. Duong, H. Lu, P. L. De Souza, M. H. Stenzel, *Biomacromolecules* **2013**, 14, 962-975.
